# Fibronectin-gelatin nanofilm coating improves dental pulp stem cell survival and differentiation in odontogenesis-mimicking organotypic 3D bilayered constructs

**DOI:** 10.3389/fdmed.2026.1763201

**Published:** 2026-02-23

**Authors:** Alexandra Jimenez-Armijo, Isaac Maximiliano Bugueno, Fadi Jerbaka, Eve Suss, Gaétan Caravello, Marzena Kawczynski, Youri Arntz, Agnès Bloch-Zupan, Varvara Gribova

**Affiliations:** 1 Institut de Génétique et de Biologie Moléculaire et Cellulaire (IGBMC), Université de Strasbourg, Illkirch, France; 2 Centre de Référence des Maladies Rares Orales et Dentaires, CRMR-O-Rares, Hôpitaux Universitaires de Strasbourg (HUS), Filière de Santé TETECOU, ERN CRANIO, Strasbourg, France; 3 Orofacial Development & Regeneration Unit, Center of Dental Medicine, Faculty of Medicine, University of Zurich, Zurich, Switzerland; 4 Faculté de Chirurgie Dentaire, Université de Strasbourg, Strasbourg, France; 5 Laboratoires de Diagnostic Génétique, Institut de Génétique Médicale D’Alsace, Hôpitaux Universitaires de Strasbourg, Strasbourg, France; 6 Biomaterials and Bioengineering, Inserm UMR_S 1121, CNRS EMR 7003, Université de Strasbourg, Strasbourg, France; 7 Accélérateur de Recherche Technologique (ART-ARNm), Inserm US55 and Laboratoire Interdisciplinaire Pour L'Innovation et la Recherche en Santé D'Orléans (LI2RSO), Thérapies Innovantes et Nanomédecine, Université D’Orléans, Orléans, France

**Keywords:** 3D models, extracellular matrix, layer-by-layer, odontogenesis, rare diseases

## Abstract

Healthcare professionals, researchers, patients and their families affected by rare diseases face many difficulties during diagnosis. A targeted diagnostic tool, using high-throughput sequencing (NGS) technologies, known as GenoDENT, used to unravel molecular diagnosis behind rare diseases with oral and dental manifestations, enabled the identification of more than 15% of variants of unknown significance (Class III - VUS), beside a high 70%–80% diagnostic rate in the analyzed patients’ cohort. VUS make diagnosis more difficult because they prevent precise correlation between genotype and phenotype. To overcome this issue, we are developing 3D *in vitro* models mimicking odontogenesis. Our first 3D models, made of odontoblast-like and ameloblast-like cells, were effective when using murine cells. They were stable over time and showed a good distinction between both used cell types. However, the formation of 3D models from human cells was less efficient, so we decided to couple the 3D model formation technique with the cell-accumulation method to produce stable 3D constructs. This process consists in covering the cells with a biomimetic artificial matrix made of fibronectin (FN) and gelatin (G). We found that FN/G coating improved viability of human dental pulp stem cells (hDPSC) in thick constructs and promoted odontoblast differentiation of hDPSC. In peripheral ameloblast-like cells, ameloblast-associated proteins such as amelogenin were detected in close contact with the odontoblast-like core. We believe that our model can be further modified to introduce patient-specific variations through gene-editing techniques like CRISPR/Cas9, for further development of new diagnostic tools applied to rare oro-dental diseases.

## Introduction

Healthcare professionals, researchers, people affected by rare diseases and their families face significant diagnostic challenges. This is the case for individuals affected by rare genetic diseases encompassing developmental dental anomalies like enamel defects in amelogenesis imperfecta (AI) and dentin abnormalities in dentinogenesis imperfecta (DI)/dentin dysplasia (DD).

Recently, a targeted genetic study on rare diseases with oral manifestations, using high-throughput sequencing technologies (GenoDENT (1–3), has allowed the identification of numerous pathogenic genetic variants (>70% of positive diagnostic yield), but also numerous variants of unknown significance (VUS) (1). The potential pathogenicity and functional impact of these VUS remain uncertain, rendering genetic diagnosis difficult. In this context, *in vitro* models of odontoblast-like and ameloblast-like cells carrying mutations (e.g., engineered using CRISPR-Cas9 technology) can be used to unravel the pathogenicity of VUS during amelogenesis and dentinogenesis. Three-dimensional (3D) models are of particular interest, since odontogenesis takes place in a 3D environment.

Tissue engineering is a powerful approach that associates cells with materials to generate tissues for various applications, from fundamental studies to tissue replacement (4). In the field of dental research, tooth regeneration is a key challenge, and several models are currently available (5). However, human odontogenesis models are rare and are usually constructed using only one cell type (6). Thus, RNA expression levels of epithelial cell markers, dental epithelial cell markers, and ameloblast markers significantly increased in hiPSC-derived ameloblast organoids as compared to 2D cultures (7). In another study,induced early ameloblast organoids were co-cultured with primary human dental pulp stem cells (hDPSC) to assess the interaction level between the two cell types and the effects on ameloblast maturation (8). This co-culture in suspension could induce AMELX expression in ameloblast organoids and induce strong DSPP expression in the odontoblast organoids. In a recent work, our team described the construction of organotypic 3D bilayered constructs made of odontoblast-like and ameloblast-like cells to mimic odontogenesis and more particularly the late bell stage (9). At this point, differentiated odontoblasts secrete an extracellular matrix mainly composed of type I collagen, known as pre-dentin. At the same time, pre-ameloblasts come into direct contact with the pre-dentin and the odontoblasts, and differentiate into ameloblasts that deposit enamel matrix proteins such as amelogenin. The method was efficient for murine cells, with a good distinction between the two cell types and good stability of the models over time. However, the formation of the constructs from human cells was less efficient: the constructs were disorganized and demonstrated reduced cell-cell adhesion (9).

In this new study, we applied the cell-accumulation method (10) to cultures of human dental pulp stem cells (hDPSC)-derived 3D constructs for the first time. We verified fibronectin and gelatin (FN/G) coating deposition and evaluated cell viability in 3D constructs made of FN/G-coated hDPSC before differentiating them into odontoblast-like cells and adding ameloblast-like cells to create organotypic 3D bilayered constructs mimicking the late bell stage cytodifferentiation of odontogenesis.

## Materials and methods

### Cell culture

Human Dental Pulp Stem Cells (hDPSC) are multipotent stem cells harvested from soft living pulp tissue inside adult teeth. hDPSC were provided (courtesy of Dr. Petros Papagerakis, from The University of Saskatchewan, Canada). The cells were grown in αMEM medium + GlutaMAX-I with 1 g/L of D-glucose and sodium pyruvate (Gibco, Thermo Fisher Scientific, Illkirch-Graffenstaden, France), supplemented with 15% fetal calf serum (FCS) and 100 U/mL penicillin and 100 mg/mL of streptomycin (this medium will be further referred to as GM) at 37°C in a humid environment with 5% CO_2_. These cells were further differentiated into odontoblast-like cells in the organotypic constructs. Human ameloblast-like cells were initially characterized as ameloblastoma cells (AM-1) and were originally obtained from a 20-year-old female, immortalized with HPV-16 vector, and clone selection was performed with 1 mg/mL G418. Cells were provided by Dr. Sylvie Babajko and Prof. Ariane Berdal (UMR-S 1333 Santé Orale, Universite Paris Cité, and, Physiopathological basis of skeletal dysplasia, Université Paris Cité, Inserm UMR-S 1163-IHU Imagine, Paris, France) and were grown in Keratinocyte SFM medium (Thermo Fisher Scientific, Illkirch-Graffenstaden, France) complemented with 1 mg/mL G418 (Roche) for clone selection and 50 μg/mL of Gentamicin at 37°C in a humidified atmosphere with 5% CO_2_.

### 2D cell cultures immunofluorescence

Immunofluorescence on 2D cell cultures was performed at room temperature after plating hDPSC and AM1 in a 24 well-plate (5 × 10^4^ cells/well) and fixing with PFA 4%. Following a 15 min PBS-Tween 0.2% treatment, cells were incubated with Bovine serum albumin (BSA) 0.5% and Triton X-100 0.1% solution in PBS (PBS-BT) for 15 min. Antibodies (primary: COL1A1: mouse anti-human, Santa Cruz, sc-293182, dilution 1/100; DSPP: rabbit anti-human, Bioss, bs-10316R, dilution 1/200; FAM83H: rabbit anti-human, Invitrogen, PA5–55094, dilution 1/100; AMELX: mouse anti-human, Santa Cruz, sc-365284, dilution 1/100; secondary: Alexa Fluor 488, donkey anti-mouse IgG ThermoFisher, A-21202, dilution 1/200; Alexa Fluor 488, donkey anti-rabbit IgG, ThermoFisher, A-21206, dilution 1/250) were incubated for 1 h, followed by incubation in phalloidin 1/1000 in PBS-1% BSA solution for 15 min. Finally, one drop 1 µg/mL of 4′,6-diamidino-2-phenylindole (DAPI) solution was added to each slide. The slides were observed using Leica Dmi 8 + Yokogawa CSU W1 - ILAS2 confocal microscope.

### Fabrication of 3D structures

The cells were detached from culture dishes using trypsin 0.25% EDTA 0.02% and washed with GM. The coating was performed as previously described (3). Briefly, the cells were resuspended in Tris-HCl buffer (Tris-HCl 50 mM pH 7.4) and subsequently incubated for 1 min with 0.04 mg/mL FN, G (Sigma-Aldrich) solutions in Tris-HCl buffer or with Tris-HCl buffer alone (rinsing step). To remove the solutions, the cells were centrifuged at 200 g for 1 min, and the supernatant was removed. After (FN/G)_4_FN nanofilms were formed, the cells were resuspended in GM at a desired concentration.

### Coating validation

**Figure 1:**
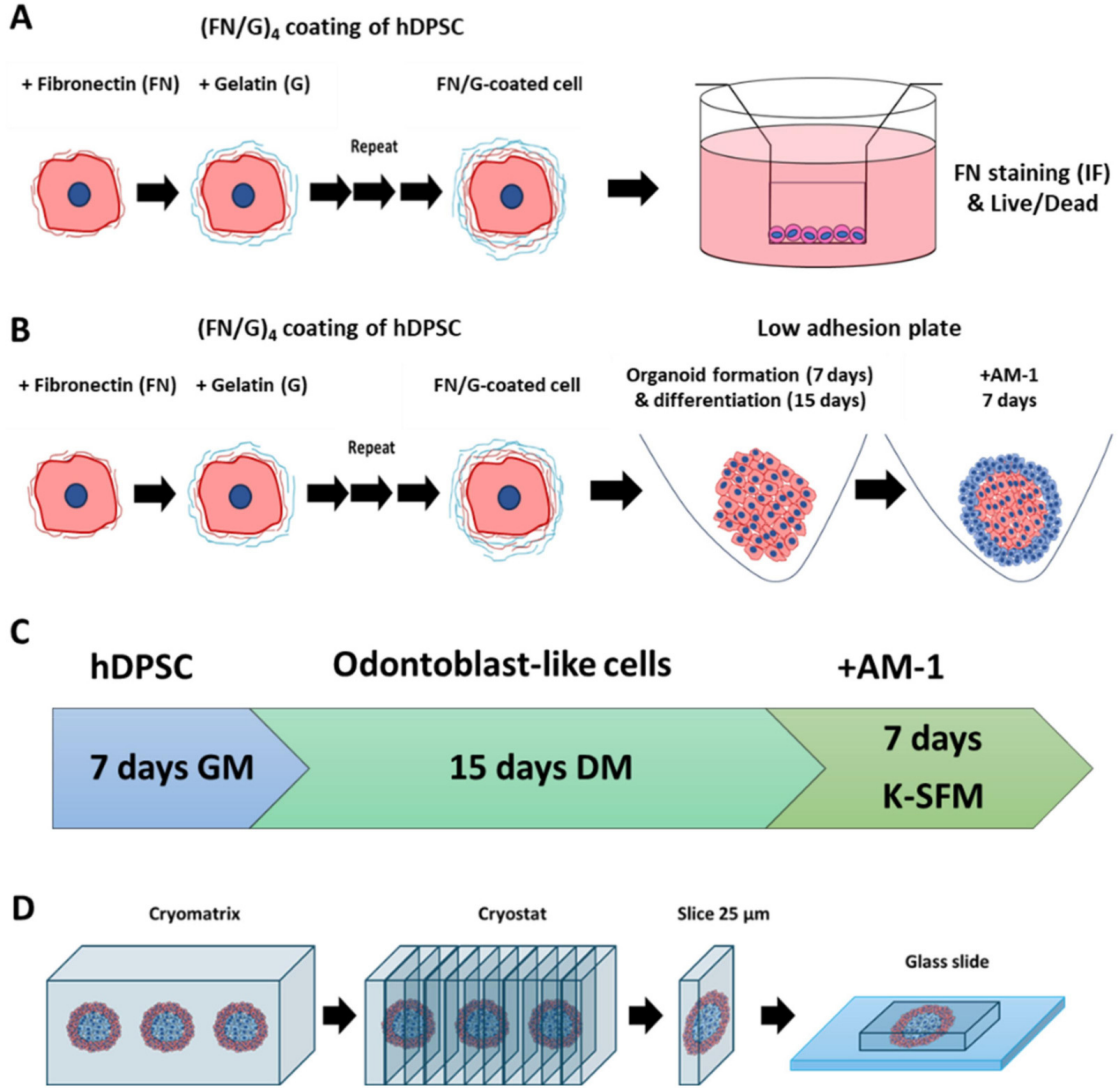
Organoid formation yand sample preparation. **(A)** Sample preparation for fibronectin/gelatin (FN/G) coating verification and cell viability assessment. **(B)** Organoid preparation from (FN/G)_4_FN-coated human dental pulp stem cells (hDPSC) and AM-1 ameloblast-like cells. **(C)** Organoid preparation timeline. GM, growth medium; DM, differentiation medium; K-SFM, keratinocyte serum-free medium. **(D)** Organoid sample preparation for fluorescent labeling.

To verify the deposition of (FN/G)_4_FN nanofilms onto the cells, 5 × 10^4^ cells were deposited into a 24-well insert with a semipermeable membrane (Corning 3470, 0.4 μm pore size) and placed into 24-well plates. One milliliter of GM was added in a 24-well plate outside the inserts. The cells were incubated for 4 h at 37 °C, and then another 1 mL of GM was added to the 24-well plate to connect the media between the inside and the outside of the insert. After 24 h the medium was removed and the cells were fixed with 4% PFA, then stained for FN (primary antibody: 1/200 rabbit anti-human, Sigma-Aldrich, F3648, secondary antibody: 1/250 Alexa Fluor 488, donkey anti-rabbit IgG, ThermoFisher, A-21206), actin, and nuclei as previously described (4). To assess the viability of the coated cells, 10^5^, 2.5 × 10^5^ cells were deposited into the inserts coated with 100 μl of 0.04 mg/mL FN at 37°C for 30 min, cultured for 4 days in GM, and submitted to Live/Dead staining according to the manufacturer's instructions (Figure 1A).

### 3D cellular organotypic culture and immunofluorescence

3D construct formation was performed using ultra-low attachment plates (PrimeSurface® 96V-shaped bottom, S-bio, Japan). First, odontoblast-like cells were cultured at a concentration of 5 × 10^3^ cells/well for 7 days in GM to allow cell attachment, then for 15 days in differentiation medium (DM). The differentiation medium was composed of the GM supplemented with 0.1 M dexamethasone, 5 mM glycerophosphate, 50 mg/mL ascorbic acid, and 10 ng/mL TGF-β1, all from Sigma-Aldrich (Merck, Darmstadt, Land de Hesse, Germany). At day 22, ameloblast-like cells were added into the wells containing differentiated odontoblast constructs at a concentration of 3 × 10^3^ cells per well and were incubated for 7 more days, ending at day 29 (Figures 1B,C). Next, the resulting structures were fixed using 4% PFA, then incorporated into a block of Epredia™ Cryomatrix™ and placed at −80°C to freeze. The block was then cut with a cryostat, and 25 µm sections were deposited on glass slides for fluorescent staining (Figure 1D).

For fluorescent staining of organoids sections, they were first treated with Triton X-100 0.1% in PBS for 15 min, then saturated with 0.1% BSA for 1 h, then incubated with primary and secondary antibodies (primary: COL1A1: mouse anti-human, Santa Cruz, sc-293182, dilution 1/100; DSPP: rabbit anti-human, Bioss, bs-10316R, dilution 1/200; FAM83H: rabbit anti-human, Invitrogen, PA5-55094, dilution 1/100; AMELX: mouse anti-human, Santa Cruz, sc-365284, dilution 1/100; secondary: Alexa Fluor 488, donkey anti-mouse IgG ThermoFisher, A-21202, dilution 1/200; Alexa Fluor 488, donkey anti-rabbit IgG, ThermoFisher, A-21206, dilution 1/250; Alexa Fluor 568, donkey anti-rabbit IgG, ThermoFisher, A-10042, dilution 1/200; Alexa Fluor 568, donkey anti-mouse IgG, ThermoFisher, A-10037, dilution 1/250) for 1 h and 30 min, respectively, and rinsed 3 times with PBS after each step. ProLong™ Diamond antifade reagent (Molecular Probes) was used for mounting. Mounted slides were observed with the Leica Dmi 8 + Yokogawa CSU W1—ILAS2 confocal microscope.

### Data analysis and statistics

Fibronectin labeling and cell viability experiment were performed 2 times, two samples per experimental condition. Cell viability values correspond to the average number of dead cells per image quantified from 10 images, and the error bars to standard deviation. Organotypic 3D construct culture and immunofluorescence results correspond to 3 independent experiments, 10 constructs per experimental condition. Odontoblast core diameter was quantified from 5 images, an average of 10 diameters measured using ImageJ software (v1.44p, NIH, Bethesda, US) was calculated. Data are reported as means ± standard deviation. Student's t-test was performed to compare coated and uncoated samples. Statistical significance was set at *p* < 0.05.

## Results

### FN/G coating maintain the viability of hDPSC in 3D cellular structures

**Figure 2:**
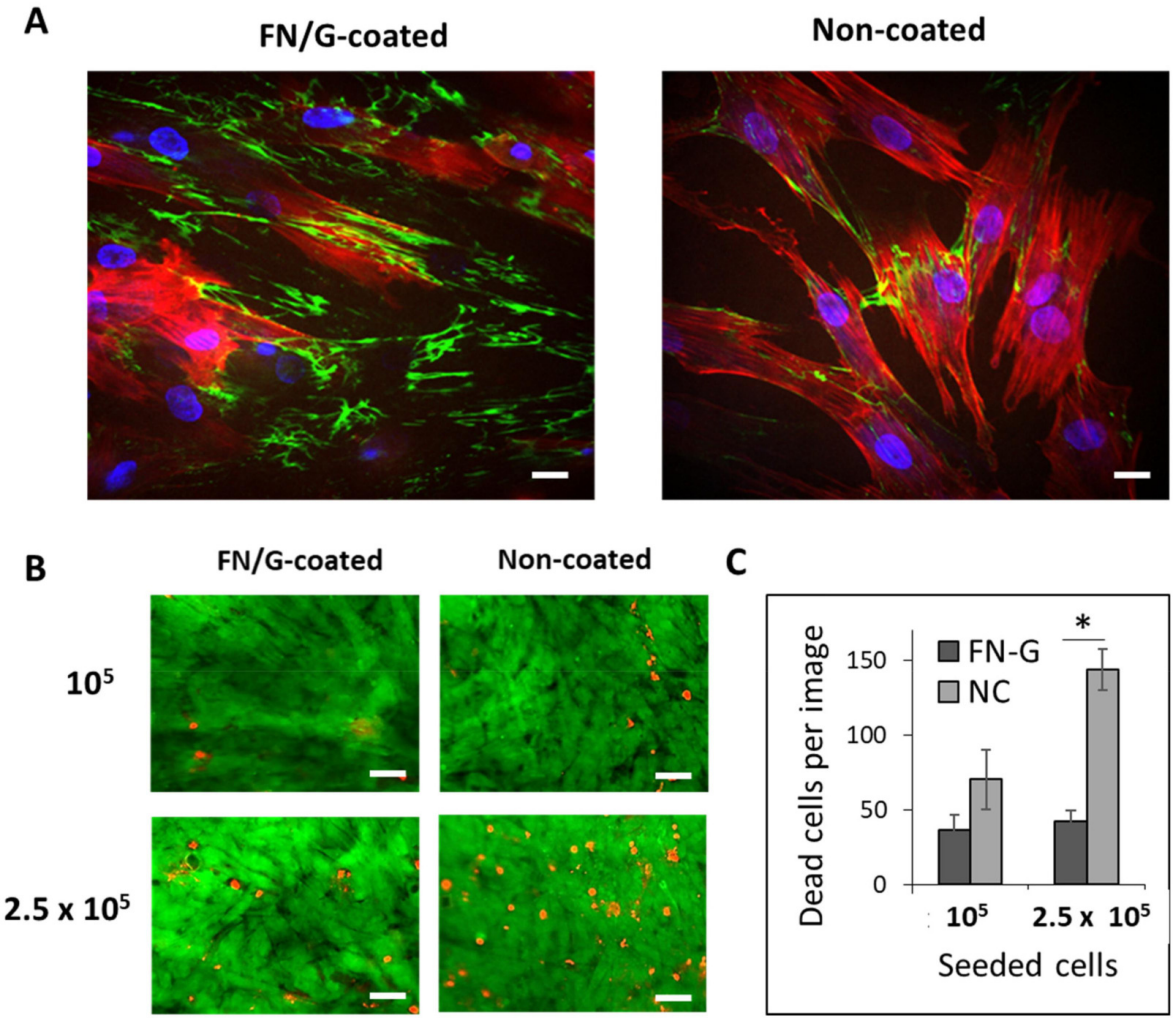
Fibronectin/gelatin (FN/G) coating and viability in 3D constructs. **(A)** FN (green), actin (red) and nuclei (blue) staining. Scale bar: 20 µm. **(B)** Live(green)/Dead (red) staining, scale bar: 100 µm. **(C)** Quantification of dead cells per image, *p < 0.01.

The cell-accumulation method, which consists of cell coating with FN/G nanomatrix, was developed by the group of Prof. Akashi and applied to different cell types, including human dermal fibroblasts (10), skeletal muscle progenitors (11) and hepatocytes (12). Here, we used it for the first time to coat hDPSC cells. The results showed the presence of FN network in monolayer samples of hDPSC coated with (FN/G)_4_FN (Figure 2A). Some FN can also be detected in uncoated samples, but in much lower quantities. This corresponds to the FN secreted by the cells.

Next, we tested cell viability in two types of constructs of different thickness (10^5^ or 2.5 × 10^5^ cells seeded into the inserts, as indicated in the Figure 1A) and found that (FN/G)_4_FN coating improved viability in thicker constructs (Figure 2B), leading to a significant decrease in the number of dead cells compared to uncoated samples (Figure 2C). These results demonstrate the importance of the cell environment for cell survival in 3D constructs and indicate that FN/G coating is appropriate to maintain the viability of hDPSC in 3D structures.

### Human ameloblast-like cells and dental pulp stem cells express amelogenesis and odontogenesis-associated proteins

**Figure 3:**
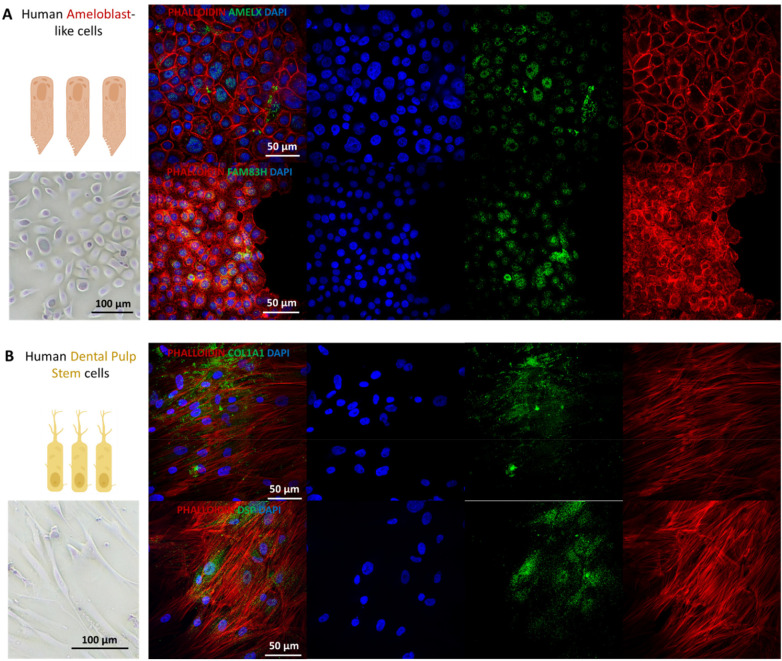
Cell morphology and protein expression in human dental pulp stem cells and ameloblast-like cells (AM1). **(A)** Immunofluorescent labeling in ameloblast-like cells AM-1 for amelogenin (AMELX) and FAM83H [**(A)**, green] and in human dental pulp stem cells for DSP-1 domain (DSP) and COL1A1 [**(B)**, green]. Actin is labelled in red (phalloidin) and nuclei in blue (DAPI). Scale bars are represented in each caption. For each immunofluorescence (IF) image, all channels are presented separately.

The basic level of expression of proteins associated with amelogenesis and odontogenesis was first assessed in 2D cultures. Type I collagen (COL1) forms most of the organic material (∼85%) in dentin, and dentin sialophosphoprotein (DSPP) is the most abundant non-collagenous protein in dentin (13). Amelogenin (AMELX) is the main enamel matrix protein, and Family with Sequence Similarity 83 Member H (FAM83H) has a role in the structural development and mineralization of enamel (14, 15). The results showed expression of these proteins in 2D cultures of AM-1 (ameloblast-like cells) and hDPSC (Figure 3), indicating that the cells were appropriate to use for formation of bilayered organoids mimicking odontogenesis. More detailed characterization of the cell types can be found in our previous work (9).

### FN/G coating favorizes odontogenic differentiation of hDPSC and gives more stability in 3D

**Figure 4:**
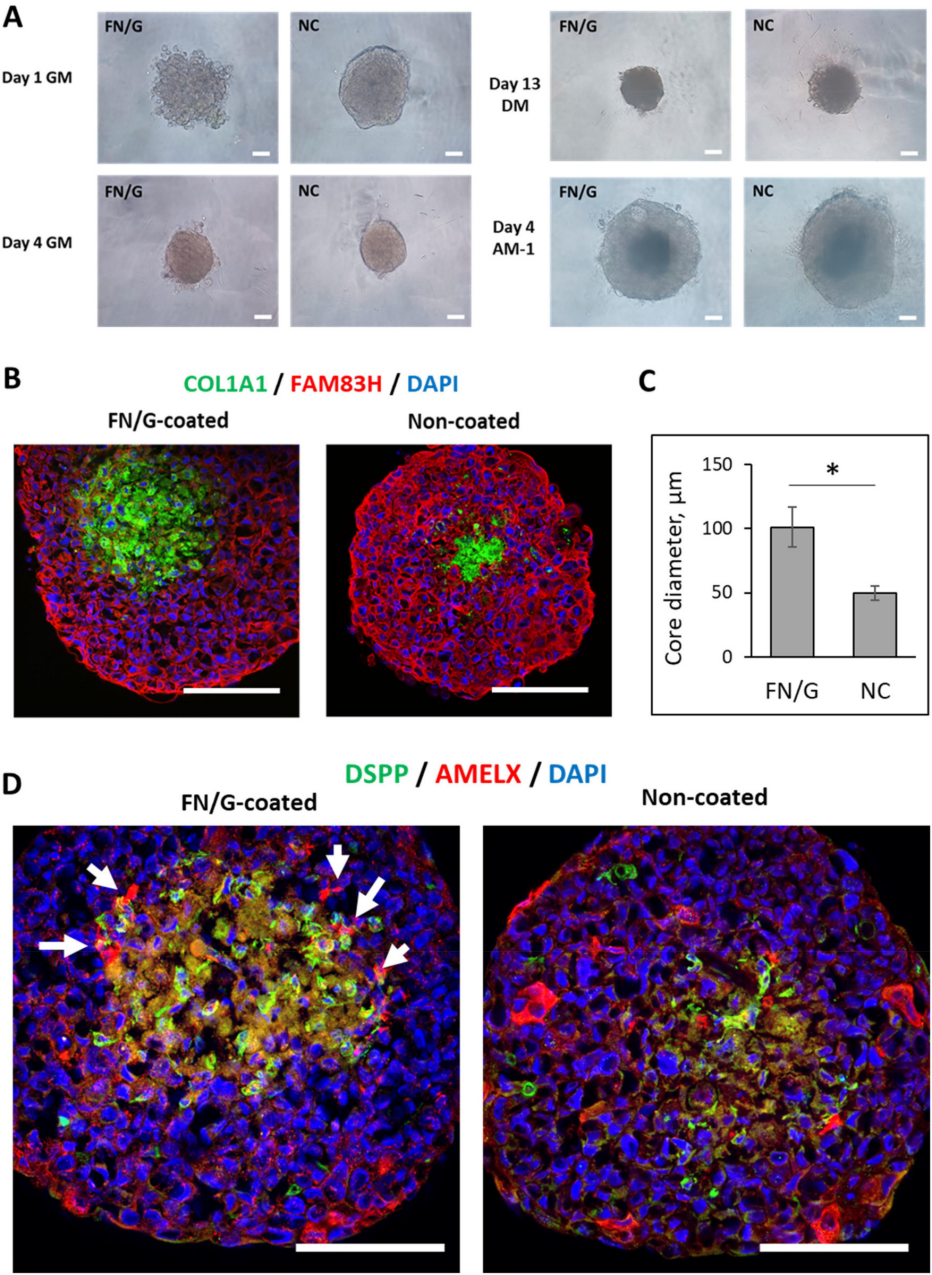
Organotypic 3D model construction and differentiation. **(A)** Phase contrast microscopy observation of bilayered construct formation from human dental pulp stem cells (hDPSC) and AM-1 cells in growth medium (GM) or under odontogenic differentiation conditions (DM). Scale bar = 50 µm. **(B)** Fluorescent labeling of bilayered construct cross-sections (7 days of hDPSC culture in GM, followed by 15 days in DM and 7 days of co-culture with AM-1). Scale bar = 100 µm. **(C)** Quantification of the odontoblast-core diameter, *p < 0.01. **(D)** Visualization of odontoblasts and ameloblast interactions. Fluorescent labeling for amelogenin (AMELX, red), dentin sialophosphoprotein (DSPP, green) and nuclei (blue) of bilayered construct cross-sections (7 days of human dental pulp stem cell culture in growth medium, followed by 15 days in odontogenic differentiation medium and 7 days of co-culture with AM-1). Scale bar = 100 µm (upper images).

Organotypic 3D bilayered construct formation from hDPSC and AM-1 cells was monitored day by day; selected time points are presented in Figure 4A. At Day 1 in GM, some differences are visible between the samples, but they disappear by Day 4. At Day 13, in DM, the structures appear dense, which is probably due to the mineralization of the hDPSC during differentiation. Finally, after AM-1 addition, at Day 4 AM-1, bilayered structures can be observed, with a denser odontoblast-based core and semi-transparent AM-1 layer (Figure 4A).

Fluorescent staining of odontoblast and ameloblast markers, COL1A1/DSPP and FAM83H/AMELX, respectively, allowed to visualize odontoblast core and the surrounding ameloblast layer in 3D construct cross-sections (Figures 4B,D). The odontoblast core was significantly larger in FN/G-coated samples (Figure 4C), suggesting that FN/G coating was favorable for odontogenic differentiation of hDPSC, making the core more stable over the 1-month culture. These results are in accordance with the observations using phase contrast: while some degradation of organoids could be observed in uncoated samples, FN/G-coated samples remained unaffected (data not shown).

The epithelial ameloblast-associated protein FAM83H showed robust staining in the ameloblast-like cell layer (Figure 4B) in both non-coated and FN/G-coated samples. While no difference of protein expression was visually observed, the variation has been detected at mRNA level (Supplementary Table S1; Supplementary Figure S1), with a 3-fold increase in *FAM83H* expression in FN/G-coated samples compared to non-coated samples, indicating that a well-differentiated and stable odontoblast core is favorable for *FAM83H* expression.

AMELX protein expression was observed in both non-coated and FN/G-coated samples. However, while it was randomly distributed in AM-1 cell layer in non-coated samples, in FN/G-coated samples it was mostly localized in contact with the odontoblast core (Figure 4D, arrows), which reflects what happens *in vivo* during odontogenesis: during enamel maturation, ameloblasts attach to the dentin surface (13). Precise molecular mechanisms of epithelio-mesenchymal crosstalk during tooth formation are difficult to study *in vitro* using 2D cell culture, therefore the development of appropriate 3D odontogenesis models can help to better understand these processes. In the study by Alghadeer et al. AMELX and ENAM were visualized at the interface between 3D co-cultured human iPSC-derived epithelial organoids and hDPSC (8). Here, we propose a different 3D system with a stable odontoblast-like core, a surrounding ameloblast-like layer and AMELX expression at the interface. As AMELX localized expression is very challenging to precisely quantify, we present only qualitative imaging results in the present work. In the future, 3D construct permeabilization will be applied to image odontoblast-ameloblast interactions in 3D and provide quantitative results. In addition, different batches of hDPSC could be tested to evaluate the potential variability between hDPSC from different donors. Another limitation of our study concerns using immortalized AM-1 cells, which could be replaced by human-induced pluripotent stem cells (hiPSCs) differentiated into human dental epithelial cells using established protocol (7).

## Conclusion

In the present study, we employed, for the first time, FN/G-coated hDPSC that were used to form organotypic 3D bilayered structures with AM-1 ameloblast-like cells after odontoblastic differentiation of hDPSC. The results emphasize the importance of the cell environment for cell viability in 3D cultures, as well as for long-term 3D culture stability. This relatively easy to construct model can become an organotypic platform for studying odontogenic differentiation in the context of rare oro-dental diseases, for instance to study patient-specific variations after gene editing to improve diagnostics.

## Data Availability

The original contributions presented in the study are included in the article/Supplementary Material, further inquiries can be directed to the corresponding authors.
