# Supplementary material for: Fibronectin-gelatin nanofilm coating improves dental pulp stem cell survival and differentiation in odontogenesis-mimicking organotypic 3D bilayered constructs
Source: Front Dent Med. 2026 Feb 23;7:1763201. doi: 10.3389/fdmed.2026.1763201 (PMC12968307; doi:10.3389/fdmed.2026.1763201)
Supplement: Supplementary file 1 [file Datasheet1.pdf]

Supporting information

Fibronectin-gelatin nanofilm coating improves dental pulp stem cell survival and differentiation in odontogenesis-mimicking organotypic 3D bilayered constructs

Alexandra Jimenez-Armijo[1,2], *Isaac Maximiliano Bugueno*[1,3], *Fadi Jerbaka*[1,2,4], *Eve Suss*[1], *Gaétan Caravello*[2,5], *Marzena Kawczynski*[1,2], *Youri Arntz*[4,6], *Agnès Bloch-Zupan*[1,2,4,*], *Varvara Gribova*[1,7,*]

[1]Institut de Génétique et de Biologie Moléculaire et Cellulaire (IGBMC), INSERM U1258, CNRS UMR7104, Université de Strasbourg, Illkirch, France
[2]Centre de Référence des maladies rares orales et dentaires, CRMR-O-Rares, Hôpitaux Universitaires de Strasbourg (HUS), Filière de santé TETECOU, ERN CRANIO, Strasbourg, France
[3]Orofacial Development & Regeneration Unit, Center of Dental Medicine, Faculty of Medicine, University of Zurich, Plattenstrasse 11, Zurich CH-8032, Switzerland.
[4]Faculté de Chirurgie Dentaire, Université de Strasbourg, Strasbourg, France
[5]Laboratoires de diagnostic génétique, Institut de Génétique Médicale d'Alsace, Hôpitaux Universitaires de Strasbourg, Strasbourg, France
[6]Biomaterials and Bioengineering, Inserm UMR_S 1121,CNRS EMR 7003, Université de Strasbourg, Strasbourg, France
[7]Accélérateur de Recherche Technologique (ART-ARNm), Inserm US55 and Laboratoire Interdisciplinaire pour l'Innovation et la Recherche en Santé d'Orléans (LI²RSO), Thérapies innovantes et Nanomédecine, Université d'Orléans, Orléans, France

* Corresponding authors

Quantitative real time PCR (RT-qPCR)

Total RNA was extracted from bilayered organoids made from non-coated or fibronectin/gelatin (FN/G)-coated human dental pulp stem cells at three time points: after 5 days of culture in growth medium (GM), after 5 days of culture in growth medium then 14 days in differentiation medium (GM ➔ DM), and after 5 days of culture in growth medium, 14 days in differentiation medium and 7 days in presence of AM-1 cells (GM ➔ DM ➔ + AM-1). Constructs were carefully collected and processed for RNA extraction using a RNeasy Plus Micro Kit according to manufacturer's instructions (Qiagen). First-strand cDNA synthesis from organoids mRNA was performed with SuperScript® IV Reverse Transcriptase (Invitrogen). LightCycler® 480 SYBR Green I Master (Roche Life Science) incorporation into amplified PCR products was detected using a RealPlex 2 qPCR Real Time PCR TermoCycler. Primer sequences (listed in Table S1) were designed using the Primer3web program. Expression was normalized to glyceraldehyde-3-phosphate dehydrogenase (GAPDH) levels. Non-coated samples were normalized to non-coated 5-day in GM (= undifferentiated) sample values, and FN/G-coated samples were normalized to FN/G-coated 5-day in GM (= undifferentiated) sample values. Tests were performed in triplicate to confirm variations. The results are presented in Figure S1.

Table S1. Primers used in RT-qPCR experience.

Gene	Forward primer	Reverse primer
FAM83H	5'-TTCGCTGAGCAGGTCCACAT	5'-AGTGAACTCAGACCAGGCCG
GAPDH	5'-CCACCCATGGCAAATTCCATGGCA	5'-TCTAGACGGCAGGTCAGGTCCACC

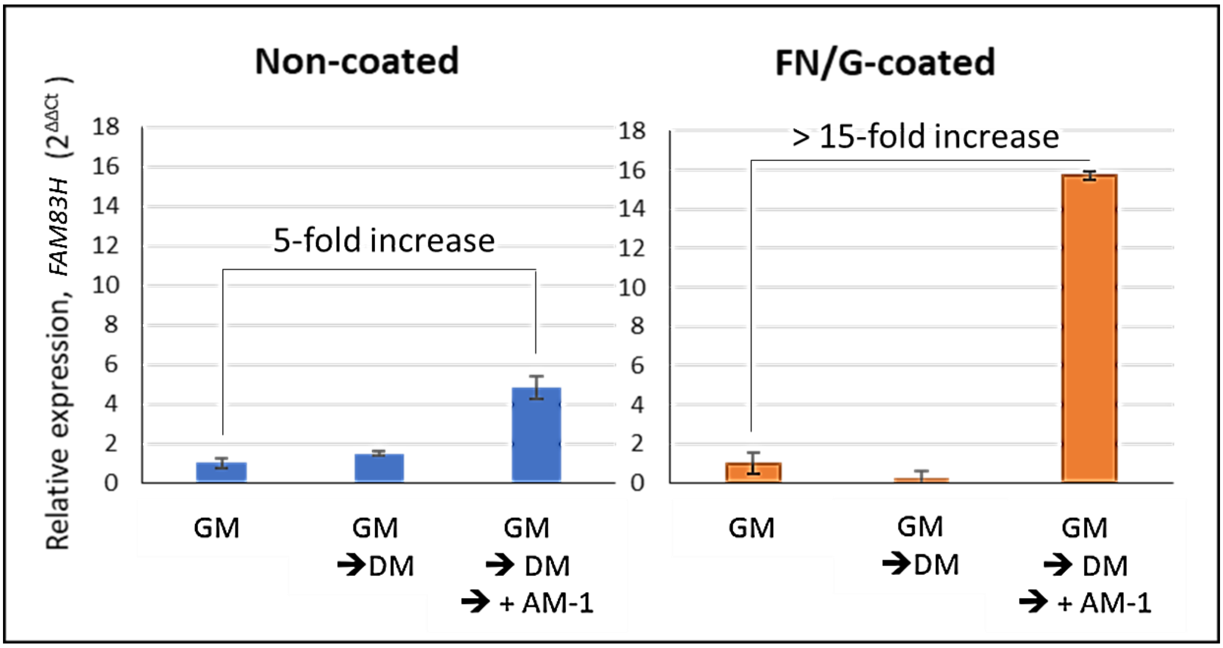

Figure S1. *FAM83H* expression was evaluated using qRT-PCR in bilayered constructs made from non-coated or fibronectin/gelatin (FN/G)-coated human dental pulp stem cells at three time points: after 5 days of culture in growth medium (GM), after 5 days of culture in growth medium then 14 days in differentiation medium (GM➔ DM), and after 5 days of culture in growth medium, 14 days in differentiation medium and 7 days in presence of AM-1 cells (GM ➔ DM ➔ + AM-1). Non-coated samples were normalized to non-coated 5-day in GM (= undifferentiated) sample values, and FN/G-coated samples were normalized to FN/G-coated 5-day in GM (= undifferentiated) sample values.
